# The neglected model validation of antimicrobial resistance transmission models – a systematic review

**DOI:** 10.1186/s13756-025-01574-x

**Published:** 2025-05-28

**Authors:** Maja L. Brinch, Andrea Palladino, Jeroen Geurtsen, Thierry Van Effelterre, Lorenzo Argante, Michael J. McConnell, Lene Christiansen, Michelle A. Pihl, Natasja K. Lund, Tine Hald

**Affiliations:** 1 Risk-Benefit, DTU National Food Institute, Kgs. Lyngby, Denmark; 2 GSK, Siena, Italy; 3 Bacterial Vaccines Discovery & Early Development, Janssen Vaccines & Prevention B.V, Leiden, The Netherlands; 4 Johnson & Johnson, Global Commercial Strategy Organization, Beerse, Belgium; 5 Department of Biological Sciences, University of Notre Dame, Notre Dame, USA

**Keywords:** Antimicrobial resistance, Transmission modelling, Interventions, Systematic review, Trace criterion

## Abstract

**Background:**

In the fight against antimicrobial resistance, mathematical transmission models have been shown as a valuable tool to guide intervention strategies in public health.

**Objective:**

This review investigates the persistence of modelling gaps identified in earlier studies. It expands the scope to include a broader range of control measures, such as monoclonal antibodies, and examines the impact of secondary infections.

**Methods:**

This review was conducted according to the PRISMA guidelines. Gaps in model focus areas, dynamics, and reporting were identified and described. The TRACE paradigm was applied to selected models to discuss model development and documentation to guide future modelling efforts.

**Results:**

We identified 170 transmission studies from 2010 to May 2022; *Mycobacterium tuberculosis* (*n* = 39) and *Staphylococcus aureus* (*n* = 27) resistance transmission were most commonly modelled, focusing on multi-drug and methicillin resistance, respectively. Forty-one studies examined multiple interventions, predominantly drug therapy and vaccination, showing an increasing trend. Most studies were population-based compartmental models (*n* = 112). The TRACE framework was applied to 39 studies, showing a general lack of description of test and verification of modelling software and comparison of model outputs with external data.

**Conclusion:**

Despite efforts to model antimicrobial resistance and prevention strategies, significant gaps in scope, geographical coverage, drug-pathogen combinations, and viral-bacterial dynamics persist, along with inadequate documentation, hindering model updates and consistent outcomes for policymakers. This review highlights the need for robust modelling practices to enable model refinement as new data becomes available. Particularly, new data for validating modelling outcomes should be a focal point in future modelling research.

**Supplementary Information:**

The online version contains supplementary material available at 10.1186/s13756-025-01574-x.

## Background

Antimicrobial resistance (AMR) is recognised by the World Health Organization (WHO) as one of the top ten global health threats [1], as it undermines the effectiveness of treatments for common infections, leading to prolonged illness, disability, higher healthcare costs, and increased mortality [2–4]. Despite the growing need, the development of new antibiotics has been limited, as they are often reserved as “last-line” treatments, reducing economic incentives for pharmaceutical companies [5]. In response, various strategies to combat AMR have been explored, including WHO’s 2021 action framework on leveraging vaccines [6], followed by a report estimating vaccines’ potential to reduce AMR and antibiotic use in both viral and bacterial infections [7]. Additionally, monoclonal antibodies (mAbs) have been proposed as preventive and therapeutic alternatives to antibiotics [8, 9]. Mathematical models have proven instrumental in understanding and addressing the complex dynamics of AMR. These models not only allow for the prediction of the potential impact of new interventions, such as new vaccines or mAbs, but also help optimise their implementation by simulating real-world scenarios and guiding evidence-based decision-making. Despite their demonstrated value, the full potential of modelling remains underutilised by public health managers and stakeholders, who could leverage these tools more effectively to inform policy development, resource allocation, and long-term strategies for combating AMR.

Existing literature has documented several systematic reviews on AMR transmission models with diverse objectives. Niewiadomska et al. (2019) reviewed population-level transmission of AMR to assess the state of research, refraining from further model evaluation [10]. In contrast, Atkins et al. (2018) examined models addressing the impact of vaccination on AMR within human populations [11]. Birkegård et al. (2018) evaluated models of AMR development and dissemination [12]. They assessed the strengths and weaknesses of the models using the TRACE framework, which comprises eight elements for documenting model testing and analysis to facilitate the creation of useful models [13].

The systematic reviews conclude that most models exhibit significant gaps in both their construction and the areas they investigate. There is a pressing need for models that integrate disease transmission and the disease burden of AMR, moving beyond mere incidence and prevalence, including economic evaluations. By employing transmission models with outcomes measured in DALYs or combining them with cost-effectiveness models, policymakers can more effectively compare and prioritize potential intervention strategies for combating antimicrobial resistance in the future. Additionally, existing reviews predominantly focus on primary bacterial infections, often neglecting viral infections. However, viral infections significantly contribute to the AMR crisis by leading to the frequent, yet ineffective, prescription of antibiotics and increasing the risk of secondary bacterial infections [14–16].

This review aims to investigate whether the modelling gaps identified by earlier reviews persist. The scope of the review is expanded to (1) Broaden the search to encompass a wider range of control measures, including monoclonal antibodies, and (2) Examine studies that model the impact of secondary infections. Additionally, the review will discuss model development and documentation by applying the TRACE paradigm to selected models, with the goal of guiding future modelling efforts.

## Methods

We conducted a systematic search and review of publications covering the modelling of AMR transmission in the human population. The full study protocol is available in the PROSPERO database (ID: CRD42022328174), which follows the Preferred Reporting Items for Systematic Reviews and Meta-analyses guidelines (PRISMA) [17].

### Search strategy and selection of papers

We systematically searched MEDLINE (Web of Science) and Scopus, see Table 1. The searches were done May 18–19, 2022. The searches were limited by publication date (any studies published from January 2010), publication type (journal article, review article, and systematic review) and English language. In addition, grey literature was systematically searched; these included reports published by the European Centre for Disease Prevention and Control (ECDC) and the World Health Organization (WHO, including Global Index Medicus).

**Table 1:** Search terms used to retrieve modelling studies

In Web of Science (MEDLINE), we performed a comprehensive search combining a core search string (Search #1) with specific areas of interest (Search #2, #3, and #4). **Search #1**: Focuses on resistance and modelling techniques. ((MHX=(Drug Resistance, Microbial OR Drug Resistance, Multiple)) AND TS = ((“mathematical” OR “dynamic” OR “transmission” OR “compartmental” OR” ODE” OR “SIR”) OR (“BoD” OR “burden of disease” OR “epidemiolog*”) AND (“model*” OR “tool”))) **Search #2**: Targets respiratory infections. TS=(“respiratory infect*” OR “viral infect*” OR “influenza” OR “RSV” OR “covid” OR “sars” OR “mers”) **Search #3**: Focuses on vaccines and monoclonal antibodies. TS=(“vaccine” OR “vaccinat*” OR “monoclonal” OR “antibod*” OR “mab*”) **Search #4**: Addresses broader control measures. TS=(“control” OR “prevention” OR “prophyl*” OR “hygiene” OR “intervention” OR “mitigation”) The final search combines the core search with the specific areas of interest: **i.e. #1 AND #2 OR #1 AND #3 OR #1 AND #4**
**Scopus**: TITLE-ABS-KEY ( ( "antimicrobial resistance" OR "antimicrobial resistant" OR "drug resistance" OR "drug resistant" OR multidrug-resistant OR "multi-drug resistant" OR "multidrug resistance" OR "multi-drug resistance" ) AND ( "mathematical" OR "dynamic" OR "transmission" OR "compartmental" OR "ODE" OR "SIR" OR "BoD" OR "burden of disease" OR "epidemiolog*" ) AND ( "model*" OR "tool" ) ) AND ( TITLE-ABS-KEY ( "control" OR "prevention" OR "prophyl*" OR "hygiene" OR "intervention" OR "mitigation" ) OR ( "vaccine" OR "vaccinat*" OR "monoclonal" OR "antibod*" OR "mab*" ) OR ( "respiratory infect*" OR "viral infect*" OR "influenza" OR "RSV" OR "covid" OR "sars" OR "mers" ) ) AND ( LIMIT-TO ( LANGUAGE , "English" ) ) AND ( LIMIT-TO ( EXACTKEYWORD , "Human" ) OR LIMIT-TO ( EXACTKEYWORD , "Humans" ) )

Publications were uploaded to the software Covidence (2022) [18] for abstract and full-text screening and subsequent data extraction of selected studies. We removed duplicate publications between the databases and grey literature. We broadly included any mathematical or computational models covering AMR in bacterial infectious disease that modelled “between-host” transmissions (i.e., population-based models), including studies that considered transmission between humans as well as involving animals or the environment. Studies were excluded if they described pharmacokinetic-pharmacodynamic models and descriptive statistical analysis. Since the review focuses on model structure, geographical restrictions were not applied.

Initially, studies were screened for eligibility based on title and abstract, and afterwards, a full-text assessment was completed. Two reviewers reviewed each selected full text. The list of publications for data extraction was agreed upon by consensus and through discussion with a third reviewer. The reference list of reviews and systematic reviews was screened for additional studies. The data extractions were carried out in Covidence. For each publication, two reviewers independently extracted data, as shown in Table 2. Any uncertainties were resolved by consensus.

**Table 2:** Description of the data extracted from the included publications

Category	Details
Disease and population description	Pathogen, resistance, infection type, setting (healthcare, community), and intervention.
Methodology	Model class (compartmental, risk assessment, burden study, etc.), model type (population vs. individual-based, deterministic vs. stochastic, and dynamic vs. static), data use, and economic evaluation.
Model description	Purpose, assumption, model outcome, strengths, limitations, and conclusion.
Model validation	Validation, calibration, and sensitivity analysis.
Metadata	Country, year of publication, programming software, funding, and possible conflicts of interest.

### Model development and documentation

We used the TRACE framework, which describes guidelines for good practice in model development and documentation [13]. The TRACE framework includes eight criteria: (1) problem formulation, (2) model description, (3) data evaluation, (4) conceptual model evaluation, (5) implementation verification, (6) model output verification, (7) model analysis and (8) model output corroboration [13]. While the full TRACE framework includes eight comprehensive criteria, we limited our assessment to models meeting criteria 6 and 7, as our primary aim was to evaluate the reliability of model outputs rather than to review all aspects of model development and documentation. This targeted approach was also taken by Birkegaard et al. (2018) and allowed us to concentrate on models with sufficient methodological transparency and rigour to support meaningful interpretation and application in the context of AMR.

## Results

**Fig. 1:**
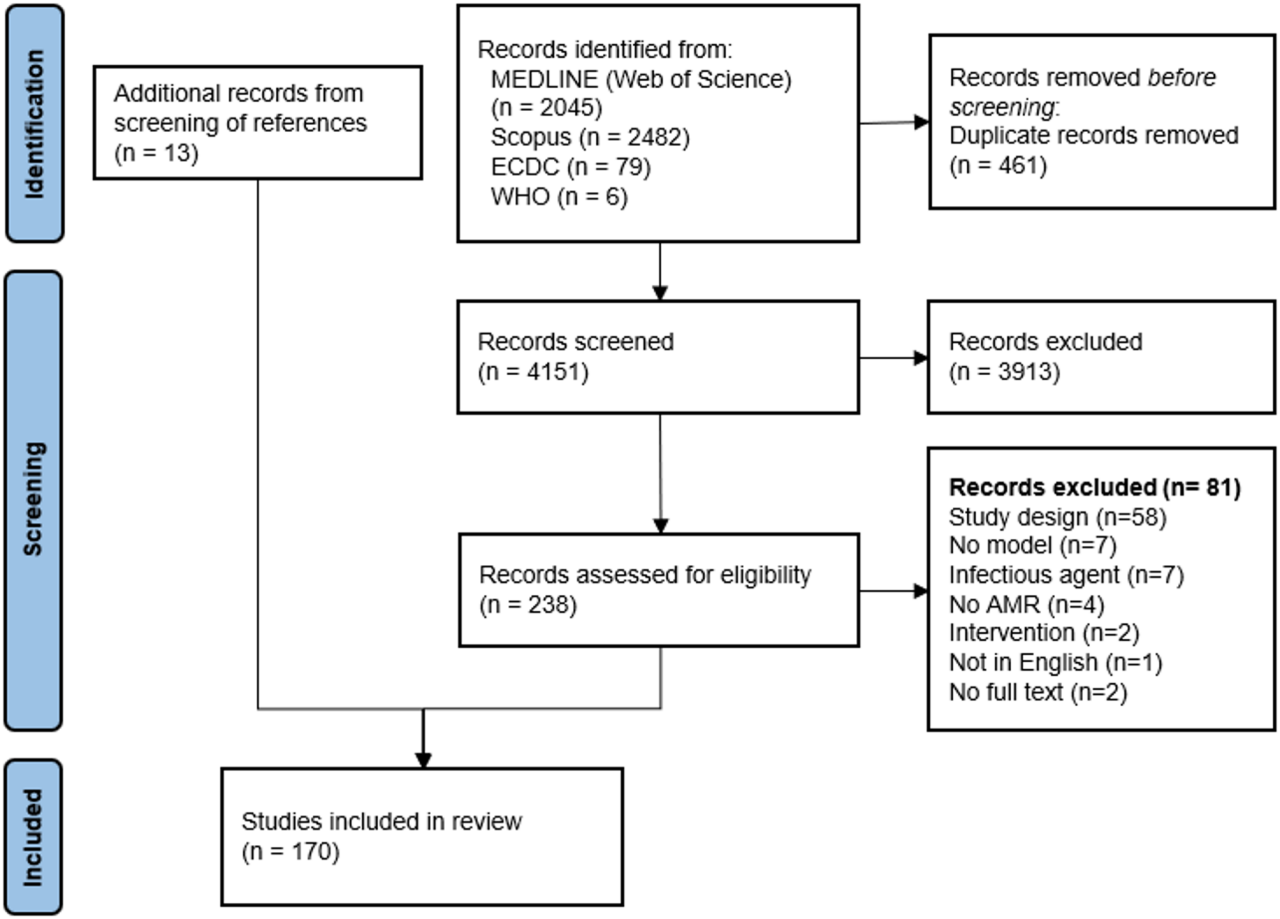
PRISMA flowchart of selection of included studies

Of 4151 articles, 3919 were excluded based on title and abstract screening, with 81 more excluded during full-text screening. The main reasons for exclusion were not relevant study designs, e.g., statistical and within-host models. An additional 13 studies were found through the reference lists screening of earlier reviews. Ultimately, 170 studies were included in the review. A summary of the exclusion process is presented in Fig. 1.

### Trends in published modelling studies

Of the 170 studies considered in the review, approximately a third of the models did not specify a particular geographic area (*n* = 56, 32.9%). In the studies that specified a geographical setting, the most modelled WHO regions were the European Region (19.4%) and the region of the Americas (17.1%). None of the studies targeted the Eastern Mediterranean Region. Some studies covered countries in two or more regions (*n* = 11, 6.5%) and five (2.9%) had a global perspective of modelling.

*Mycobacterium tuberculosis* (*n* = 39, 22.9%), *Staphylococcus aureus* (*n* = 27, 15.9%), and *Streptococcus pneumoniae* (*n* = 14, 8.2%) were the most modelled pathogens. In total, 22 papers modelled more than one pathogen (12.9%), whereas 24 papers (14.1%) did not specify the pathogen of interest in their model. The most frequent drug-pathogen combinations were multidrug-resistant tuberculosis, methicillin-resistant *S. aureus* (MRSA), extended spectrum beta-lactamase (ESBL) resistant *Escherichia coli*, ESBL and carbapenem-resistant *Enterobacterales*, and vancomycin-resistant *Enterococci.* For a detailed list of pathogens studied in each publication, see Table S1.

**Fig. 2:**
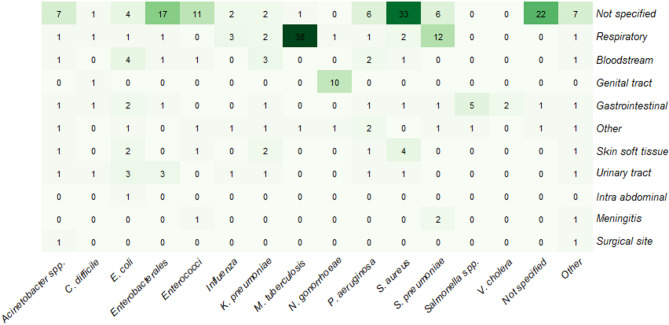
Heat map of modelled pathogen across infection site. Studies modelling multiple pathogens are split into specific pathogen/infection site combinations

The studies modelled colonisation (*n* = 53, 31.1%), infection (*n* = 84, 49.4%) or both (*n* = 33, 19.4%). Notably, only one study specifically modelled secondary infections [19]. For models specifying both colonisation and infection, only 11 models specified the site of infection (6.5%). The most common types of infections modelled were respiratory infections (*n* = 49, 28.8). Nine of the studies modelled more than one infection site. However, the site of infection was not specified in approximately half of the studies (*n* = 89, 52.4%), whereas some studies (*n* = 22, 12.9%) did not specify the pathogen either. Figure 2 present a heatmap of modelled pathogens across infection sites.

**Fig. 3:**
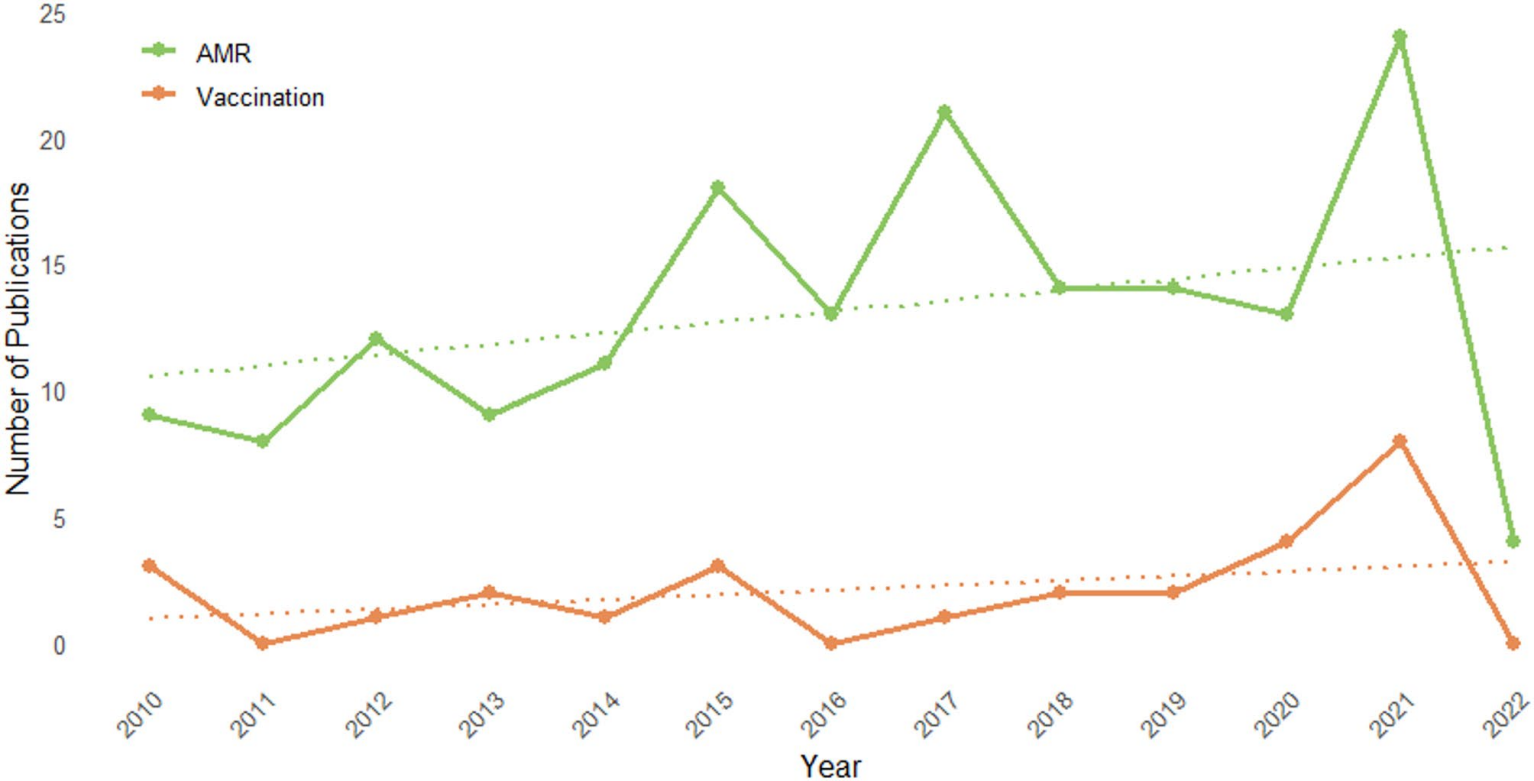
Yearly number of included modelling publications. The Figure compares the total number of included AMR modelling studies with included studies modelling vaccination as an intervention (January 2010 to May 2022)

A large fraction of the studies modelled transmission in the community (*n* = 72, 42.9%) or in healthcare (*n* = 60, 35.7%) setting, where 26 were restricted to intensive care units (ICU). A smaller fraction of the studies modelled the transmission between the community and the healthcare system (*n* = 23, 13.7%) or between animals and humans (*n* = 3, 1.8%). Lastly, 12 (7.1%) did not specify the setting where they modelled transmission. A total of 127 (74.7%) studies modelled interventions, of which 41 investigated more than one intervention. Six did not specify the type of intervention. Different strategies for drug therapy were the most investigated types of intervention (*n* = 67). These included reduced use, mixing or cycling of antibiotics, among others. Drug therapy interventions were followed by vaccination (*n* = 27) and hand hygiene (*n* = 21). There was an increasing trend in the annual publication of AMR transmission models, and a growing trend is seen in modelling vaccines as an intervention against AMR (Fig. 3). However, vaccines were mainly modelled targeting respiratory infections.

### Model structure and dynamics

Most studies were population-based models (*n* = 134, 78.8%), where 112 were built as compartmental models. Individual-based modelling was used in 32 studies (18.8%). Only a few studies included both types of models (*n* = 4, 2.4%); see Table S2. Models were predominantly deterministic (*n* = 107, 62.9%) compared to stochastic modelling (*n* = 48, 28.2%). Several studies used both deterministic and stochastic modelling approaches (*n* = 15, 8.8%). Most models were dynamic (*n* = 138, 81%) as opposed to static (*n* = 32, 19%).

Studies included in the review had several different model outcomes (mortality, prevalence, incidence, fraction of resistance, etc.). Lastly, only a small fraction of studies included an economic evaluation in the model (*n* = 26, 15.3%). Vaccination and other interventions were frequently modelled as various scenarios based on theoretical or hypothetical effects by adjusting specific model parameters, such as reduced antibiotic usage, decreased transmission due to improved hygiene, or variations in vaccine characteristics like duration of protection, efficacy, and uptake.

Model calibration was done for 69 (40.6%) of the models, whereas 115 (67.6.%) of the studies performed a sensitivity analysis of the model parameters. Only a third of the models mentioned a validation process, either of model input or model output, applying external data (*n* = 56, 32.9%).

### Model development and documentation

**Fig. 4:**
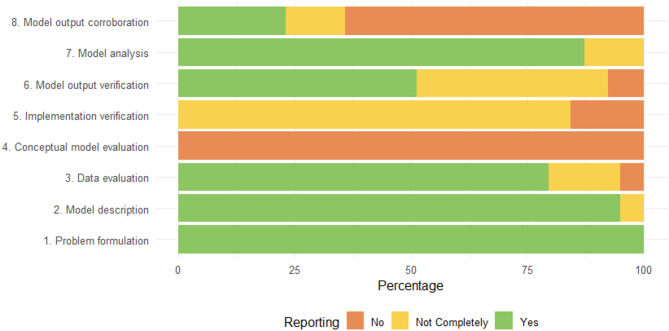
Evaluation of the fulfilment of TRACE of studies reporting validation of the model and sensitivity analysis

Of the 170 included papers, 39 studies reported validation of the model *and* conducted a sensitivity analysis, thus complying with TRACE criteria 6 and 7. None of the papers fulfilled all eight TRACE criteria, as none of the studies described code validation, criterion 5 (Implementation verification). The reviewed papers generally lacked a description of software implementation and code references. Even though the studies mention internal validation of the model outputs, 48.7% (*n* = 19) showed insufficient or no information on the validation process. Around 36% (*n* = 14) of the studies made a comparison of the model predictions with external data (criterion 8); however, some did not provide sufficient information on the comparison (Fig. 4; Table 3).

**Table 3:** Evaluation of the fulfilment of TRACE of studies reporting validation of the model and sensitivity analysis. The table does not include problem formulation (criterion 1) and implementation verification (criterion 5) as all studies meet criterion 1, and none fulfill criterion 5

Study	Model description	Data evaluation	Conceptual model evaluation	Model output verification	Model analysis	Model output corroboration
Almagor, 2018 [20]	Yes	No	No	Not completely	Not completely	No
Bartsch, 2016 [21]	Yes	Yes	Yes	No	Yes	No
Cen, 2017 [22]	Yes	No	Yes	Yes	Yes	No
Chen, 2019 [23]	Yes	Yes	Yes	Yes	Yes	No
Cilloni, 2020 [24]	Yes	Yes	Yes	Not completely	Yes	No
Colijn, 2020 [25]	Yes	No	Yes	Yes	Not completely	No
Deeny, 2015 [26]	Yes	Yes	Yes	Not completely	Yes	Yes
DiRuscio, 2019 [27]	Yes	Yes	Yes	Not completely	Yes	Yes
Duan, 2021 [28]	Yes	Yes	Yes	Not completely	Yes	No
Fu, 2021 [29]	Yes	Yes	Yes	Yes	Yes	No
Han, 2021 [30]	Yes	No	Yes	Yes	Not completely	No
Harris, 2017 [31]	Not completely	Yes	Yes	Yes	Yes	Yes
Ho, 2016 [32]	Yes	Yes	Not completely	Not completely	Yes	No
Hogea, 2013 [33]	Yes	Yes	Yes	Yes	Yes	No
Hughes, 2017 [34]	Yes	Not completely	Yes	Not completely	Yes	No
Kachalov, 2021 [35]	Yes	Yes	Yes	Not completely	Yes	Not completely
Kardaś-Słoma, 2017 [36]	Yes	Not completely	Yes	Not completely	Yes	Not completely
Laager, 2021 [37]	Yes	Yes	Yes	Yes	Yes	No
Lee, 2013 [38]	Not completely	Yes	Not completely	Not completely	Yes	No
Lee, 2016 [39]	Yes	Yes	Not completely	Yes	Yes	Yes
Liao, 2012 [40]	Yes	No	Yes	Yes	Yes	Not completely
Liechti 2017 [41]	Yes	No	Not completely	Not completely	Yes	No
Machlaurin, 2020 [42]	Yes	Yes	Not completely	Yes	Yes	No
Menzies, 2018 [43]	Yes	Yes	Yes	Not completely	Yes	Yes
Mponponsuo, 2022 [44]	Yes	Yes	Yes	No	Yes	Not completely
Murray, 2022 [45]	Yes	Yes	Yes	Yes	Yes	No
Opatowski, 2021 [46]	Yes	No	Yes	Yes	Yes	No
Ozawa, 2021 [47]	Yes	Yes	Yes	Not completely	Yes	No
Panchanathan, 2010 [48]	Yes	No	Yes	Yes	Yes	No
Pečerska, 2021 [49]	Yes	No	Yes	Yes	Not completely	Yes
Suen, 2014 [50]	Yes	Yes	Yes	Yes	Yes	No
Suthar, 2014 [51]	Yes	Yes	No	Yes	Yes	Yes
Talaminos, 2016 [52]	Yes	Yes	Yes	Not completely	Yes	Yes
Toth, 2021 [53]	Yes	Yes	Yes	Not completely	Not completely	Not completely
Vilches, 2019 [54]	Yes	Yes	Yes	Not completely	Yes	Yes
Wang, 2013 [55]	Yes	Yes	Not completely	Yes	Yes	No
Weerasuriya, 2021 [56]	Yes	Yes	Yes	No	Yes	No
Yahdi, 2012 [57]	Yes	No	Yes	Yes	Yes	No
Zacher, 2019 [58]	Yes	No	Yes	Yes	Yes	No

## Discussion

This systematic review explored gaps in mathematical models studying AMR bacterial infection transmission in humans. The reviewed publications ranged from simple deterministic to complex stochastic models across various settings. Models were primarily compartmental, population-based, dynamic, and deterministic. These findings are consistent with earlier reviews [10, 12]. Many models incorporated hypothetical scenarios (e.g., absence of specific pathogens, resistance profiles, settings, or infection sites). This is likely due to the scarcity of available data.

Murray et al. [59] estimated that Sub-Saharan Africa carries the highest burden of AMR. Nevertheless, the models in this review primarily focused on high-income regions, such as Europe and North America, where surveillance data are more readily accessible. Studies have demonstrated that AMR is widespread in low- and middle-income countries [60, 61], highlighting the urgent need to establish surveillance systems and expand access to diagnostics and antimicrobial susceptibility testing capabilities. Key pathogens contributing to AMR-associated deaths include *E. coli*, *S. aureus*, and *K. pneumoniae*, highlighted by the European Antimicrobial Resistance Collaborators [4] and the WHO’s priority list for antibiotic research and development [62], which also includes *M. tuberculosis* as a critical priority [63]. Our review concurred with these high-impact pathogens regarding the number of modelled studies (e.g., *M. tuberculosis* and *S. aureus*). However, significant modelling gaps are evident for *Acinetobacter baumannii*, *Pseudomonas aeruginosa*, and community-acquired infections like *Salmonella* spp, *Campylobacter* spp, and *Helicobacter pylori*. While some studies modelled resistant *Enterobacterales*, future studies should preferably model at the species level to incorporate specific dynamics and better assess intervention impacts on relevant target groups.

Few studies encompassed both colonisation and infection stages, along with specific infection sites (e.g., respiratory, bloodstream, or urinary tract infections). Disparities in the burden across infection sites are demonstrated, with over 1,500,000 deaths attributed to AMR for lower respiratory infections compared to less than 250,000 for skin infections [59]. Future models could gain from explicitly addressing colonisation and infection, focusing on specific infection sites. This approach would provide a more realistic representation of the impact of infection prevention and control strategies. The primary challenge for modellers, and a key limiting factor of many models, is the scarcity of available data. Many models rely on a blend of estimates or secondary data from other studies. To ensure their efficacy, validating these models by comparing model outcomes with independent real-world data is imperative. It is crucial to improve data sharing practices to address the challenge of data scarcity. Enhanced collaboration between clinicians and modellers can facilitate the collection of specific data on intervention effectiveness, significantly boosting model accuracy and reliability. Improved access to diagnostics in low-resource settings can enhance data availability and representativeness, enabling the development of models that more accurately reflect transmission routes specific to these environments. For instance, inadequate sanitation may lead to increased transmission from environmental and water sources [64]. Establishing and continuously updating standardised protocols for data collection and sharing can ensure more comprehensive and representative datasets. Furthermore, using stochastic modelling frameworks can better account for uncertainty in model outputs, providing a more nuanced understanding of intervention impacts.

Only one study investigated the burden associated with secondary bacterial infections following primary viral infections [19] despite the well-established role of secondary bacterial infections in driving antibiotic use and contributing to the overall AMR burden [65, 66]. This notable gap warrants critical attention. One possible explanation for the limited modelling in this area is the lack of comprehensive data on co-morbidities. It is well-recognised that bacterial co-infections disproportionately affect vulnerable individuals with underlying conditions, such as chronic respiratory diseases, diabetes, cardiovascular disorders, or immunosuppressive disorders [65, 67]. However, data linking these co-morbidities with infection outcomes, antibiotic use, and resistance patterns are often sparse or inconsistently reported. This lack of granularity hampers efforts to model high-risk populations accurately and to assess targeted interventions effectively. Model complexity is likely adding to this modelling gap. Modelling the transmission dynamics of viral and secondary bacterial infections is complex due to the need to integrate, e.g., multiple transmission pathways, co-infection interactions, spatial-temporal factors, and control measures [68]. The public health significance of such modelling efforts is, however, essential for predicting infection spread and evaluating intervention strategies, but they require careful consideration of the various biological and environmental factors involved.

A considerable number of the reviewed models lacked proper descriptions of internal and external validation, which are essential steps in establishing the credibility and reliability of a model’s results. Internal validation involves assessing whether the model behaves as expected, given its structure and input data. This can include model calibration, where parameters are adjusted to align outputs with observed data, and face validation, where experts review the model’s logic and behaviour to determine whether it reasonably represents the real-world system being modelled. These approaches help ensure internal consistency and that the model is performing as intended. In contrast, external validation tests the model’s predictive accuracy by comparing its outputs to independent, real-world data not used during model development. For example, this could involve validating projected trends in antimicrobial resistance against surveillance data or comparing the predicted effects of vaccination strategies with observed outcomes from actual public health interventions. Without both internal and external validation, a model’s utility for informing policy and public-health decisions remains limited. Still, a very limited number of the assessed papers described both internal and external validation properly. However, to adhere to best practices, the methods for the validations should be articulated. Comprehensive descriptions of models are necessary for replication by other researchers. While many studies presented parameters through flowcharts or tables, the models were often inadequately described for replication. None of the assessed models provided information on criterion 5 – implementation verification. It is plausible that this was conducted but not explicitly reported. To address this issue, there is a critical need for open-source code to ensure transparency, facilitate replication, and enable thorough verification of implementation. By making the code publicly accessible, researchers can validate findings, reproduce results, and build upon existing work to ensure the creation of useful models with reliable findings for decision-makers.

Studies showcased a diverse array of model outputs, with many addressing one or several health outcomes like mortality, prevalence, incidence, or the fraction of resistance. Other studies focused either on risk metrics or the disease’s reproduction number. This diversity complicates result comparison across pathogens, infection sites, and settings. Only a few studies employed the well-established disease burden measure, DALYs. This measure is advantageous as it incorporates years lost to premature mortality and years lived with disabilities [69]. DALYs facilitate comparing burdens between infections and settings to identify where intervention may be more impactful. In addition, it is possible to compare across infectious and non-communicable diseases. While there have been many attempts to estimate short-term AMR transmission, there is a gap in modelling long-term projections for AMR reduction. Models covering the potential long-term effects of reduced antibiotic selective pressure on a population level when implementing interventions are, consequently, recommended.

The review underscores a strong emphasis on modelling the impact of various interventions against AMR, particularly regarding drug therapy (e.g., reduced use, mixing, or cycling of antibiotics). Furthermore, numerous models explored the potential of vaccines in alleviating the burden. An increasing trend has been seen since Atkins and colleagues’ review in 2018 [11]. This trend is partly driven by increasing recognition of the role vaccines can play in reducing the need for antibiotics by preventing infections in the first place and thereby slowing the emergence and spread of resistant pathogens. A key driver of this momentum is the World Health Organization. Since the publication of WHO’s global action plan on antimicrobial resistance [70], several strategies and frameworks have been tailored to the role of vaccines in both viral and bacterial infections, with a focus on expanding vaccine utilisation, advancing development and amplifying knowledge sharing [6, 71–73]. Mathematical models have proven valuable for illustrating vaccine impact [11, 74]. Combined with economic assessments, these models can provide crucial insights to support decision-making and advance vaccine initiatives. While alternative infection prevention strategies are imperative, a modelling gap was observed regarding monoclonal antibodies (mAbs), which have demonstrated efficacy in combating various viral pathogens in humans and may also contribute to controlling bacterial infections, including those associated with AMR [9].

Although this review shows a clear focus on AMR transmission in humans, a few models explore the connection with the veterinary, agricultural, or environmental sectors. The One Health framework’s importance in combating rising AMR levels is increasingly being underscored and is a key component of the strategic framework on AMR presented by the Quadripartite Joint Secretariat on AMR [75]. Researchers should encourage interdisciplinary research, develop standardised methodologies for harmonising data collection, and adopt integrative, multi-directional models across sectors to enhance models that integrate One Health. These efforts will significantly improve our understanding of the complex interactions in resistance transmission, leading to more effective strategies for combating antimicrobial resistance.

One limitation of this review is the restriction of the literature search to English publications from only two databases (Scopus and Medline), which, while comprehensive and widely used, may not capture the full breadth of relevant publications on modelling AMR. Relying solely on these databases may have led to the exclusion of studies indexed elsewhere, such as in specialised repositories (e.g., Embase, Web of Science, or preprint servers), or within grey literature, such as reports from national public health agencies. As a result, the comprehensiveness of the review may be limited, particularly in capturing interdisciplinary or emerging research from non-traditional sources.

Additionally, the selection of specific keywords to structure the search strategy, while necessary to maintain focus, may have inadvertently excluded relevant studies that used alternative terminology or phrasing. For example, models addressing AMR might be embedded within broader studies on infectious disease dynamics, vaccination strategies, or health economics without explicitly referencing “antimicrobial resistance” in the title or abstract. Such omissions could lead to an underrepresentation of certain modelling approaches or innovative methodologies, potentially biasing the review toward more conventional or well-documented studies.

Lastly, some papers may have been overlooked for the TRACE evaluation if validation or sensitivity analysis was not elucidated. Despite these limitations, this review provides a current overview of existing models examining AMR disease burden and transmission.

## Conclusion

Despite the increasing efforts to model the transmission of antimicrobial resistance and prevention strategies, significant gaps remain. These include the scope of modelling, geographical coverage, drug-pathogen combinations, and the dynamics between viral and bacterial infections. Additionally, thorough model documentation is often lacking, which hinders the ability to update models and produce consistently comparable outcomes to guide policymakers.

This review underscores the critical importance of adhering to sound modelling practices to facilitate model refinement and updates as new data becomes accessible. Particularly, obtaining new data for validating modelling outcomes should be a focal point in future modelling research.

## Electronic supplementary material

Below is the link to the electronic supplementary material.

Supplementary Material 1

## Data Availability

No datasets were generated or analysed during the current study.
