# Supplementary material for: The neglected model validation of antimicrobial resistance transmission models – a systematic review
Source: Antimicrob Resist Infect Control. 2025 May 28;14:59. doi: 10.1186/s13756-025-01574-x (PMC12121249; doi:10.1186/s13756-025-01574-x)
Supplement: Supplementary file 1 — Supplementary Material 1 [file 13756_2025_1574_MOESM1_ESM.docx]

## Supplementary material to “The neglected model validation of antimicrobial resistance transmission models – a systematic review”

**Table of contents**

Supplementary Table 1: Included studies divided by pathogens.

Supplementary Table 2: Included studies divided by model class.

### Table S1: Included studies divided by pathogens.

| **Pathogen** | **Number of studies** | **Reference** |
| --- | --- | --- |
| *M. tuberculosis* | 39 | (1–39) |
| *S. aureus* | 27 | (40–66) |
| Not specified | 24 | (67–90) |
| More than one pathogen | 22 | (91–112) |
| *S. pneumoniae* | 14 | (113–126) |
| *N. gonorrhoeae* | 11 | (127–137) |
| *Enterobacterales* | 10 | (138–147) |
| *Enterococci* | 5 | (148–152) |
| *E. coli* | 4 | (153–156) |
| *Salmonella* species | 4 | (157–160) |
| *Acinetobacter* species | 3 | (161–163) |
| Influenza | 2 | (164,165) |
| *P. aeruginosa* | 2 | (166,167) |
| *C. difficile* | 1 | (168) |
| *K. pneumoniae* | 1 | (169) |
| *V. cholerae* | 1 | (170) |

Table S2: Included studies divided by model class.

| **Model class** | **Reference** |
| --- | --- |
| Compartment model | (1,2,4–9,11,13–17,19–24,26–35,37–40,43,45–50,52,54–57,59–67,69,70,73,75–87,90,91,98,99,101–103,105–108,110,112,115–118,120–122,125,126,128,130,131,133–135,137,139,141,142,144,145,147,149,150,152–154,156–161,163–167,170) |
| Agent based model | (3,41,51,53,58,72,89,93–95,100,104,109,114,123,138,146,148) |
| Risk assessment | (10,36,68,97,113,129,143,151,162) |
| Decision tree | (12,25,124,132,140,155) |
| Burden study | (92,96,168) |
| Network analysis | (74,111) |
| Agent based and Compartment model | (42) |
| Agent based model and Decision tree | (119) |
| Compartment and Decision tree | (136) |
| Compartment model and Risk assessment | (18) |
| Compartment model and Network analysis | (88) |

## References

1. Cilloni L, Kranzer K, Stagg HR, Arinaminpathy N. Trade-offs between cost and accuracy in active case finding for tuberculosis: A dynamic modelling analysis. PLoS Med. 2020 Dec 2;17(12).

2. Denegre AA, Myers K, Fefferman NH. Impact of Strain Competition on Bacterial Resistance in Immunocompromised Populations. Antibiotics (Basel). 2020 Mar 1;9(3).

3. Espindolaa AL, Varugheseb M, Laskowskic M, Shoukatc A, Heffernand JM, Moghadas SM. Strategies for halting the rise of multidrug resistant TB epidemics: assessing the effect of early case detection and isolation. Int Health. 2017 Mar 1;9(2):80–90.

4. Fofana MO, Shrestha S, Knight GM, Cohen T, White RG, Cobelens F, et al. A Multistrain Mathematical Model To Investigate the Role of Pyrazinamide in the Emergence of Extensively Drug-Resistant Tuberculosis. Antimicrob Agents Chemother. 2017 Mar 1;61(3).

5. Fu H, Lewnard JA, Frost I, Laxminarayan R, Arinaminpathy N. Modelling the global burden of drug-resistant tuberculosis avertable by a post-exposure vaccine. Nat Commun. 2021 Dec 1;12(1).

6. Goscé L, Abou Jaoude GJ, Kedziora DJ, Benedikt C, Hussain A, Jarvis S, et al. Optima TB: A tool to help optimally allocate tuberculosis spending. PLoS Comput Biol. 2021 Sep 1;17(9).

7. Han WM, Mahikul W, Pouplin T, Lawpoolsri S, White LJ, Pan-Ngum W. Assessing the impacts of short-course multidrug-resistant tuberculosis treatment in the Southeast Asia Region using a mathematical modeling approach. PLoS One. 2021 Mar 1;16(3).

8. Hickson RI, Mercer GN, Lokuge KM. A metapopulation model of tuberculosis transmission with a case study from high to low burden areas. PLoS One. 2012 Apr 4;7(4).

9. Jabbari A, Castillo-Chavez C, Nazari F, Song B, Kheiri H. A Two-strain TB model with multiple latent stages. Mathematical Biosciences and Engineering. 2016 Aug 1;13(4):741–85.

10. Jakab Z, Acosta CD, Kluge HH, Dara M. Consolidated Action Plan to Prevent and Combat Multidrug- and Extensively Drug-resistant Tuberculosis in the WHO European Region 2011-2015: Cost-effectiveness analysis. Tuberculosis. 2015 Jun 1;95(S1):S212–6.

11. Kendall EA, Azman AS, Cobelens FG, Dowdy DW. MDR-TB treatment as prevention: The projected population-level impact of expanded treatment for multidrug-resistant tuberculosis. PLoS One. 2017 Mar 1;12(3).

12. Kendall EA, Malhotra S, Cook-Scalise S, Denkinger CM, Dowdy DW. Estimating the impact of a novel drug regimen for treatment of tuberculosis: A modeling analysis of projected patient outcomes and epidemiological considerations. BMC Infect Dis. 2019 Sep 9;19(1).

13. Knight GM, Zimic M, Funk S, Gilman RH, Friedland JS, Grandjean L. The relative fitness of drug-resistant Mycobacterium tuberculosis: A modelling study of household transmission in Peru. J R Soc Interface. 2018;15(143).

14. Kuddus MA, Meeha MT, Whit LJ, McBryd ES, Adekunl AI. Modeling drug-resistant tuberculosis amplification rates and intervention strategies in Bangladesh. PLoS One. 2020 Jul 1;15(7).

15. Kuddus MA, Meehan MT, Sayem MA, McBryde ES. Scenario analysis for programmatic tuberculosis control in Bangladesh: a mathematical modelling study. Sci Rep. 2021 Dec 1;11(1).

16. Kuddus MA, McBryde ES, Adekunle AI, White LJ, Meehan MT. Mathematical analysis of a two-strain tuberculosis model in Bangladesh. Sci Rep. 2022 Dec 1;12(1).

17. Li BY, Shi WP, Zhou CM, Zhao Q, Diwan VK, Zheng X Bin, et al. Rising challenge of multidrug-resistant tuberculosis in China: A predictive study using Markov modeling. Infect Dis Poverty. 2020 Jun 8;9(1).

18. Liao CM, Lin YJ. Assessing the transmission risk of multidrug-resistant Mycobacterium tuberculosis epidemics in regions of Taiwan. Int J Infect Dis. 2012 Oct;16(10).

19. Machlaurin A, Dolk FCK, Setiawan D, van der Werf TS, Postma MJ. Cost-Effectiveness Analysis of BCG Vaccination against Tuberculosis in Indonesia: A Model-Based Study. Vaccines (Basel). 2020 Dec 1;8(4):1–14.

20. McBryde ES, Meehan MT, Doan TN, Ragonnet R, Marais BJ, Guernier V, et al. The risk of global epidemic replacement with drug-resistant Mycobacterium tuberculosis strains. International Journal of Infectious Diseases. 2017 Mar 1;56:14–20.

21. Mehra M, Cossrow N, Kambili C, Underwood R, Makkar R, Potluri R. Assessment of tuberculosis burden in China using a dynamic disease simulation model. International Journal of Tuberculosis and Lung Disease. 2013 Sep 1;17(9):1186–94.

22. Menzies NA, Cohen T, Lin HH, Murray M, Salomon JA. Population health impact and cost-effectiveness of tuberculosis diagnosis with Xpert MTB/RIF: a dynamic simulation and economic evaluation. PLoS Med. 2012 Nov;9(11).

23. Menzies NA, Cohen T, Hill AN, Yaesoubi R, Galer K, Wolf E, et al. Prospects for tuberculosis elimination in the United States: Results of a transmission dynamic model. Am J Epidemiol. 2018 Sep 1;187(9):2011–20.

24. Otoo D, Osman S, Poku SA, Donkoh EK. Dynamics of Tuberculosis (TB) with Drug Resistance to First-Line Treatment and Leaky Vaccination: A Deterministic Modelling Perspective. Comput Math Methods Med. 2021;2021.

25. Oxlade O, Piatek A, Vincent C, Menzies D. Modeling the impact of tuberculosis interventions on epidemiologic outcomes and health system costs. BMC Public Health. 2015 Feb 4;15(1).

26. Pečerska J, Kühnert D, Meehan CJ, Coscollá M, de Jong BC, Gagneux S, et al. Quantifying transmission fitness costs of multi-drug resistant tuberculosis. Epidemics. 2021 Sep 1;36.

27. Sachdeva KS, Raizada N, Gupta RS, Nair SA, Denkinger C, Paramasivan CN, et al. The potential impact of up-front drug sensitivity testing on India’s epidemic of multi-drug resistant tuberculosis. PLoS One. 2015 Jul 1;10(7).

28. Salje H, Andrews JR, Deo S, Satyanarayana S, Sun AY, Pai M, et al. The importance of implementation strategy in scaling up Xpert MTB/RIF for diagnosis of tuberculosis in the Indian health-care system: a transmission model. PLoS Med. 2014;11(7).

29. Salvatore PP, Kendall EA, Seabrook D, Brown J, Durham GH, Dowdy DW. Projecting the impact of variable MDR-TB transmission efficiency on long-term epidemic trends in South Africa and Vietnam. Sci Rep. 2019 Dec 1;9(1).

30. Sharma A, Hill A, Kurbatova E, van der Walt M, Kvasnovsky C, Tupasi TE, et al. Estimating the future burden of multidrug-resistant and extensively drug-resistant tuberculosis in India, the Philippines, Russia, and South Africa: a mathematical modelling study. Lancet Infect Dis. 2017 Jul 1;17(7):707–15.

31. Shrestha S, Knight GM, Fofana M, Cohen T, White RG, Cobelens F, et al. Drivers and trajectories of resistance to new first-line drug regimens for tuberculosis. Open Forum Infect Dis. 2014 Sep 1;1(2).

32. Suen SC, Bendavid E, Goldhaber-Fiebert JD. Disease control implications of India’s changing multi-drug resistant tuberculosis epidemic. PLoS One. 2014 Mar 7;9(3).

33. Trauer JM, Denholm JT, McBryde ES. Construction of a mathematical model for tuberculosis transmission in highly endemic regions of the Asia-pacific. J Theor Biol. 2014 Oct 7;358:74–84.

34. Trauer JM, Denholm JT, Waseem S, Ragonnet R, McBryde ES. Scenario Analysis for Programmatic Tuberculosis Control in Western Province, Papua New Guinea. Am J Epidemiol. 2016 Jun 15;183(12):1138–48.

35. Ugwu KO, Onah IS, Mbah GC, Ezeonu IM. Rifampicin resistance patterns and dynamics of tuberculosis and drug-resistant tuberculosis in Enugu, South Eastern Nigeria. J Infect Dev Ctries. 2020 Sep 1;14(9):1011–8.

36. Vesga JF, Hallett TB, Reid MJA, Sachdeva KS, Rao R, Khaparde S, et al. Assessing tuberculosis control priorities in high-burden settings: a modelling approach. Lancet Glob Health. 2019 May 1;7(5):e585–95.

37. Weerasuriya CK, Harris RC, Quaife M, McQuaid CF, White RG, Gomez GB. Affordability of Adult Tuberculosis Vaccination in India and China: A Dynamic Transmission Model-Based Analysis. Vaccines (Basel). 2021 Mar 1;9(3).

38. Weerasuriya CK, Harris RC, McQuaid CF, Bozzani F, Ruan Y, Li R, et al. The epidemiologic impact and cost-effectiveness of new tuberculosis vaccines on multidrug-resistant tuberculosis in India and China. BMC Med. 2021 Dec 1;19(1).

39. Yu Y, Shi Y, Yao W. Dynamic model of tuberculosis considering multi-drug resistance and their applications. Infect Dis Model. 2018 Jan 1;3:362–72.

40. Agusto FB. Optimal control of methicillin-resistant Staphylococcus aureus transmission in hospital settings. Appl Math Model. 2016 Apr 1;40(7–8):4822–43.

41. Barnes S, Golden B, Wasil E. MRSA Transmission Reduction Using Agent-Based Modeling and Simulation. https://doi.org/101287/ijoc11000386. 2010 Apr 15;22(4):635–46.

42. Caudill L, Lawson B. A unified inter-host and in-host model of antibiotic resistance and infection spread in a hospital ward. J Theor Biol. 2017 May 21;421:112–26.

43. Laager M, Cooper BS, Eyre DW. Probabilistic modelling of effects of antibiotics and calendar time on transmission of healthcare-associated infection. Scientific Reports 2021 11:1. 2021 Nov 1;11(1):1–11.

44. Chamchod F, Ruan S. Modeling methicillin-resistant Staphylococcus aureus in hospitals: Transmission dynamics, antibiotic usage and its history. Theor Biol Med Model. 2012 Jun 27;9(1):1–14.

45. Chamchod F, Ruan S. Modeling the spread of methicillin-resistant Staphylococcus aureus in nursing homes for elderly. PLoS One. 2012 Jan 6;7(1).

46. D’Agata EMC, Webb GF, Pressley J. Rapid Emergence of Co-colonization with Community-acquired and Hospital-Acquired Methicillin-Resistant Staphylococcus aureus Strains in the Hospital Setting. Math Model Nat Phenom. 2010 Jan;5(3):76–93.

47. Deeny SR, Worby CJ, Tosas Auguet O, Cooper BS, Edgeworth J, Cookson B, et al. Impact of mupirocin resistance on the transmission and control of healthcare-associated MRSA. J Antimicrob Chemother. 2015 Dec 1;70(12):3366–78.

48. Ding W, Webb GF. Optimal control applied to community-acquired methicillin-resistant Staphylococcus aureus in hospitals. J Biol Dyn. 2017 Mar 17;11:65–78.

49. Ruscio F Di, Guzzetta G, Bjørnholt JV, Leegaard TM, Moen AEF, Merler S, et al. Quantifying the transmission dynamics of MRSA in the community and healthcare settings in a low-prevalence country. Proc Natl Acad Sci U S A. 2019;116(29):14599–605.

50. Hall IM, Barrass I, Leach S, Pittet D, Hugonnet S. Transmission dynamics of methicillin-resistant Staphylococcus aureus in a medical intensive care unit. J R Soc Interface. 2012 Oct 7;9(75):2639–52.

51. Harris AD, Morgan DJ, Pineles L, Perencevich EN, Barnes SL. Deconstructing the relative benefits of a universal glove and gown intervention on MRSA acquisition. Journal of Hospital Infection. 2017 May 1;96(1):49–53.

52. Hogea C, Van Effelterre T, Acosta CJ. A basic dynamic transmission model of Staphylococcus aureus in the US population. Epidemiol Infect. 2014;142(3):468–78.

53. Kardaś-Słoma L, Boëlle PY, Opatowski L, Brun-Buisson C, Guillemot D, Temime L. Impact of antibiotic exposure patterns on selection of community-associated methicillin-resistant Staphylococcus aureus in hospital settings. Antimicrob Agents Chemother. 2011 Oct;55(10):4888–95.

54. Kardas-Słoma L, Boëlle PY, Opatowski L, Guillemot D, Temime L. Antibiotic reduction campaigns do not necessarily decrease bacterial resistance: The example of methicillin-resistant Staphylococcus aureus. Antimicrob Agents Chemother. 2013 Sep;57(9):4410–6.

55. Kouyos RD, zur Wiesch PA, Bonhoeffer S. On being the right size: The impact of population size and stochastic effects on the evolution of drug resistance in hospitals and the community. PLoS Pathog. 2011 Apr;7(4).

56. Lei H, Jones RM, Li Y. Quantifying the relative impact of contact heterogeneity on MRSA transmission in ICUs-a modelling study. BMC Infect Dis. 2020 Jan 3;20(1).

57. Melese ZT, Mwalili SM, Orwa GO. Threshold dynamics of the Transmission of Antibiotic-Resistant Infections. BioSystems. 2018 Sep 1;171:80–92.

58. Milazzo L, Bown JL, Eberst A, Phillips G, Crawford JW. Modelling of Healthcare Associated Infections: A study on the dynamics of pathogen transmission by using an individual-based approach. Comput Methods Programs Biomed. 2011 Nov;104(2):260–5.

59. Panchanathan SS, Petitti DB, Fridsma DB. The development and validation of a simulation tool for health policy decision making. J Biomed Inform. 2010 Aug;43(4):602–7.

60. Pei S, Liljeros F, Shaman J. Identifying asymptomatic spreaders of antimicrobial-resistant pathogens in hospital settings. Proc Natl Acad Sci U S A. 2021 Sep 14;118(37).

61. Plipat N, Spicknall IH, Koopman JS, Eisenberg JNS. The dynamics of methicillin-resistant Staphylococcus aureus exposure in a hospital model and the potential for environmental intervention. BMC Infect Dis. 2013 Dec 17;13(1).

62. Robotham J V., Graves N, Cookson BD, Barnett AG, Wilson JA, Edgeworth JD, et al. Screening, isolation, and decolonisation strategies in the control of meticillin resistant Staphylococcus aureus in intensive care units: cost effectiveness evaluation. BMJ. 2011 Oct 15;343(7827).

63. Tang A, Kwok KO, Wei VWI, Chen H, Wong SYS, Tam WWS. Synergistic Effect of Combination Interventions for Methicillin-Resistant Staphylococcus aureus Transmission Control in Nursing Homes: A Computation Modelling Evaluation with Heterogeneous Contact Mixing. Antibiotics (Basel). 2021 Mar 1;10(3):1–12.

64. Tekle YI, Nielsen KM, Liu J, Pettigrew MM, Meyers LA, Galvani AP, et al. Controlling Antimicrobial Resistance through Targeted, Vaccine-Induced Replacement of Strains. PLoS One. 2012 Dec 5;7(12).

65. Wang J, Wang L, Magal P, Wang Y, Zhuo J, Lu X, et al. Modelling the transmission dynamics of meticillin-resistant Staphylococcus aureus in Beijing Tongren hospital. Journal of Hospital Infection. 2011 Dec;79(4):302–8.

66. Wang X, Panchanathan S, Chowell G. A Data-Driven Mathematical Model of CA-MRSA Transmission among Age Groups: Evaluating the Effect of Control Interventions. PLoS Comput Biol. 2013;9(11).

67. Beams AB, Toth DJA, Khader K, Adler FR. Harnessing Intra-Host Strain Competition to Limit Antibiotic Resistance: Mathematical Model Results. Bull Math Biol. 2016 Sep 1;78(9):1828–46.

68. Bell G, MacLean C. The Search for “Evolution-Proof” Antibiotics. Trends Microbiol. 2018 Jun 1;26(6):471–83.

69. Blanquart F, Lehtinen S, Lipsitch M, Fraser C. The evolution of antibiotic resistance in a structured host population. J R Soc Interface. 2018;15(143).

70. Cen X, Feng Z, Zheng Y, Zhao Y. Bifurcation analysis and global dynamics of a mathematical model of antibiotic resistance in hospitals. J Math Biol. 2017 Dec 1;75(6–7):1463–85.

71. Chowa K, Wanga X, Curtiss I, Castillo-Chavez C. Evaluating the efficacy of antimicrobial cycling programmes and patient isolation on dual resistance in hospitals. J Biol Dyn. 2011 Jan;5(1):27–43.

72. Almagor J, Temkin E, Benenson I, Fallach N, Carmeli Y. The impact of antibiotic use on transmission of resistant bacteria in hospitals: Insights from an agent-based model. PLoS One. 2018 May 1;13(5).

73. Haber M, Levin BR, Kramarz P. Antibiotic control of antibiotic resistance in hospitals: a simulation study. BMC Infect Dis. 2010 Aug 25;10.

74. Karkada UH, Adamic LA, Kahn JM, Iwashyna TJ. Limiting the spread of highly resistant hospital-acquired microorganisms via critical care transfers: a simulation study. Intensive Care Med. 2011 Oct;37(10):1633–40.

75. Knipl D, Röst G, Moghadas SM. Population dynamics of epidemic and endemic states of drug-resistance emergence in infectious diseases. PeerJ. 2017;2017(1).

76. Kouyos RD, Abel zur Wiesch P, Bonhoeffer S. Informed switching strongly decreases the prevalence of antibiotic resistance in hospital wards. PLoS Comput Biol. 2011;7(3).

77. Kunkel A, Colijn C, Lipsitch M, Cohen T. How could preventive therapy affect the prevalence of drug resistance? Causes and consequences. Philosophical Transactions of the Royal Society B: Biological Sciences. 2015;370(1670).

78. Levin BR, Baquero F, Johnsen PJ. A model-guided analysis and perspective on the evolution and epidemiology of antibiotic resistance and its future. Vol. 19, Current Opinion in Microbiology. Elsevier Ltd; 2014. p. 83–9.

79. Liechti JI, Leventhal GE, Bonhoeffer S. Host population structure impedes reversion to drug sensitivity after discontinuation of treatment. PLoS Comput Biol. 2017 Aug 1;13(8):e1005704.

80. Meehan MT, Cocks DG, Trauer JM, McBryde ES. Coupled, multi-strain epidemic models of mutating pathogens. Math Biosci. 2018 Feb 1;296:82–92.

81. Obolski U, Stein GY, Hadany L. Antibiotic Restriction Might Facilitate the Emergence of Multi-drug Resistance. PLoS Comput Biol. 2015 Jun 25;11(6):1004340.

82. Park AW, Haven J, Kaplan R, Gandon S. Refugia and the evolutionary epidemiology of drug resistance. Biol Lett. 2015 Nov 1;11(11).

83. Porco TC, Gao D, Scott JC, Shim E, Enanoria WT, Galvani AP, et al. When Does Overuse of Antibiotics Become a Tragedy of the Commons? PLoS One. 2012 Dec 7;7(12).

84. Qu L, Pan Q, Gao X, He M. Population Dynamics of Patients with Bacterial Resistance in Hospital Environment. Comput Math Methods Med. 2016;2016.

85. Robinson M, Stilianakis NI. A model for the emergence of drug resistance in the presence of asymptomatic infections. Math Biosci. 2013 Jun;243(2):163–77.

86. Saddler CA, Wu Y, Valckenborgh F, Tanaka MM. Epidemiological control of drug resistance and compensatory mutation under resistance testing and second-line therapy. Epidemics. 2013 Dec;5(4):164–73.

87. Uecker H, Bonhoeffer S. Modeling antimicrobial cycling and mixing: Differences arising from an individual-based versus a population-based perspective. Math Biosci. 2017 Dec 1;294:85–91.

88. Van Den Dool C, Haenen A, Leenstra T, Wallinga J. The Role of Nursing Homes in the Spread of Antimicrobial Resistance Over the Healthcare Network. Infect Control Hosp Epidemiol. 2016 Feb 12;37(7):761–7.

89. Wares JR, Lawson B, Shemin D, D’Agata EMC. Evaluating infection prevention strategies in out-patient dialysis units using agent-based modeling. PLoS One. 2016 May 1;11(5).

90. Xiao Y, Brauer F, Moghadas SM. Can treatment increase the epidemic size? J Math Biol. 2016 Jan 1;72(1–2):343–61.

91. Althouse BM, Bergstrom TC, Bergstrom CT. A public choice framework for controlling transmissible and evolving diseases. Proc Natl Acad Sci U S A. 2010 Jan 26;107(SUPPL. 1):1696–701.

92. Murray CJ, Ikuta KS, Sharara F, Swetschinski L, Robles Aguilar G, Gray A, et al. Global burden of bacterial antimicrobial resistance in 2019: a systematic analysis. Lancet. 2022 Feb 12;399(10325):629–55.

93. Barnes SL, Morgan DJ, Harris AD, Carling PC, Thom KA. Preventing the transmission of multidrug-resistant organisms: modeling the relative importance of hand hygiene and environmental cleaning interventions. Infect Control Hosp Epidemiol. 2014 Sep 1;35(9):1156–62.

94. Barnes SL, Rock C, Harris AD, Cosgrove SE, Morgan DJ, Thom KA. The Impact of Reducing Antibiotics on the Transmission of Multidrug-Resistant Organisms. Infect Control Hosp Epidemiol. 2017 Jun 1;38(6):663–9.

95. Bartsch SM, Wong KF, Mueller LE, Gussin GM, McKinnell JA, Tjoa T, et al. Modeling Interventions to Reduce the Spread of Multidrug-Resistant Organisms Between Health Care Facilities in a Region. JAMA Netw Open. 2021 Aug 4;4(8).

96. Cassini A, Högberg LD, Plachouras D, Quattrocchi A, Hoxha A, Simonsen GS, et al. Attributable deaths and disability-adjusted life-years caused by infections with antibiotic-resistant bacteria in the EU and the European Economic Area in 2015: a population-level modelling analysis. Lancet Infect Dis. 2019 Jan 1;19(1):56–66.

97. Crellen T, Turner P, Pol S, Baker S, Nguyen TNT, Stoesser N, et al. Transmission dynamics and control of multidrug-resistant klebsiella pneumoniae in neonates in a developing country. Elife. 2019 Dec 1;8.

98. D’Agata EMC, Horn MA, Ruan S, Webb GF, Wares JR. Efficacy of infection control interventions in reducing the spread of multidrug-resistant organisms in the hospital setting. PLoS One. 2012 Feb 20;7(2).

99. Gao D, Lietman TM, Porco TC. Antibiotic resistance as collateral damage: the tragedy of the commons in a two-disease setting. Math Biosci. 2015 May 1;263:121–32.

100. Glushchenko OE, Prianichnikov NA, Olekhnovich EI, Manolov AI, Tyakht A V., Starikova E V., et al. VERA: agent-based modeling transmission of antibiotic resistance between human pathogens and gut microbiota. Bioinformatics. 2019 Oct 1;35(19):3803–11.

101. Gurieva T, Dautzenberg MJD, Gniadkowski M, Derde LPG, Bonten MJM, Bootsma MCJ. The Transmissibility of Antibiotic-Resistant Enterobacteriaceae in Intensive Care Units. Clin Infect Dis. 2018 Feb 15;66(4):489–93.

102. Kachalov VN, Nguyen H, Balakrishna S, Salazar-Vizcaya L, Sommerstein R, Kuster SP, et al. Identifying the drivers of multidrug-resistant Klebsiella pneumoniae at a European level. PLoS Comput Biol. 2021 Jan 29;17(1).

103. Kanyiri CW, Luboobi L, Kimathi M. Application of Optimal Control to Influenza Pneumonia Coinfection with Antiviral Resistance. Comput Math Methods Med. 2020;2020.

104. Khader K, Thomas A, Huskins WC, Stevens V, Keegan LT, Visnovsky L, et al. Effectiveness of contact precautions to prevent transmission of methicillin-resistant staphylococcus aureus and vancomycin-resistant enterococci in intensive care units. Clinical Infectious Diseases. 2021 Jan 15;72:S42–9.

105. Olesen SW, Lipsitch M, Grad YH. The role of “spillover” in antibiotic resistance. Proc Natl Acad Sci U S A. 2020 Nov 17;117(46):29063–8.

106. Ong KM, Phillips MS, Peskin CS. A mathematical model and inference method for bacterial colonization in hospital units applied to active surveillance data for carbapenem-resistant enterobacteriaceae. PLoS One. 2020 Nov 1;15(11):e0231754.

107. Paul P, Slayton RB, Kallen AJ, Walters MS, Jernigan JA. Modeling Regional Transmission and Containment of a Healthcare-associated Multidrug-resistant Organism. Clin Infect Dis. 2020 Feb 1;70(3):388–94.

108. Seigal A, Mira P, Sturmfels B, Barlow M. Does Antibiotic Resistance Evolve in Hospitals? Bull Math Biol. 2017 Jan 1;79(1):191–208.

109. Slayton RB, Toth D, Lee BY, Tanner W, Bartsch SM, Khader K, et al. Vital signs: Estimated effects of a coordinated approach for action to reduce antibiotic-resistant infections in health care facilities - United States. American Journal of Transplantation. 2015 Nov 1;15(11):3002–7.

110. Squire MM, Igusa T, Siddiqui S, Sessel GK, Squire EN. Cost-Effectiveness of Multifaceted Built Environment Interventions for Reducing Transmission of Pathogenic Bacteria in Healthcare Facilities. Health Environments Research and Design Journal. 2019 Apr 1;12(2):147–61.

111. Vilches TN, Bonesso MF, Guerra HM, Fortaleza CMCB, Park AW, Ferreira CP. The role of intra and inter-hospital patient transfer in the dissemination of heathcare-associated multidrug-resistant pathogens. Epidemics. 2019 Mar 1;26:104–15.

112. Joice R, Lipsitch M. Targeting Imperfect Vaccines against Drug-Resistance Determinants: A Strategy for Countering the Rise of Drug Resistance. PLoS One [Internet]. 2013 Jul 25 [cited 2021 Oct 25];8(7):e68940. Available from: https://journals.plos.org/plosone/article?id=10.1371/journal.pone.0068940

113. Barnes CE, MacIntyre CR. Risk modelling the mortality impact of antimicrobial resistance in secondary pneumococcal pneumonia infections during the 2009 influenza pandemic. Int J Infect Dis. 2019 Aug 1;85:1–6.

114. Chen HH, Stringer A, Eguale T, Rao GG, Ozawa S. Impact of Antibiotic Resistance on Treatment of Pneumococcal Disease in Ethiopia: An Agent-Based Modeling Simulation. Am J Trop Med Hyg. 2019;101(5):1042–53.

115. Cobey S, Baskerville EB, Colijn C, Hanage W, Fraser C, Lipsitch M. Host population structure and treatment frequency maintain balancing selection on drug resistance. J R Soc Interface. 2017 Aug 1;14(133).

116. Colijn C, Corander J, Croucher NJ. Designing ecologically optimized pneumococcal vaccines using population genomics. Nat Microbiol. 2020 Mar 1;5(3):473–85.

117. Davies NG, Flasche S, Jit M, Atkins KE. Modeling the effect of vaccination on selection for antibiotic resistance in Streptococcus pneumoniae. Sci Transl Med. 2021 Aug 11;13(606):8690.

118. De Cellès MD, Pons-Salort M, Varon E, Vibet MA, Ligier C, Letort V, et al. Interaction of Vaccination and Reduction of Antibiotic Use Drives Unexpected Increase of Pneumococcal Meningitis. Scientific Reports 2015 5:1. 2015 Jun 11;5(1):1–11.

119. Lu E, Chen HH, Zhao H, Ozawa S. Health and economic impact of the pneumococcal conjugate vaccine in hindering antimicrobial resistance in China. Proc Natl Acad Sci U S A. 2021 Mar 30;118(13).

120. Maher MC, Alemayehu W, Lakew T, Gaynor BD, Haug S, Cevallos V, et al. The fitness cost of antibiotic resistance in streptococcus pneumoniae: Insight from the field. PLoS One. 2012 Jan 17;7(1).

121. Obolski U, Lourenço J, Thompson C, Thompson R, Gori A, Gupta S. Vaccination can drive an increase in frequencies of antibiotic resistance among nonvaccine serotypes of Streptococcus pneumoniae. Proc Natl Acad Sci U S A. 2018 Mar 20;115(12):3102–7.

122. Opatowski L, Mandel J, Varon E, Boëlle PY, Temime L, Guillemot D. Antibiotic dose impact on resistance selection in the community: A mathematical model of β-lactams and streptococcus pneumoniae dynamics. Antimicrob Agents Chemother. 2010 Jun;54(6):2330–7.

123. Ozawa S, Chen HH, Rao GG, Eguale T, Stringer A. Value of pneumococcal vaccination in controlling the development of antimicrobial resistance (AMR): Case study using DREAMR in Ethiopia. Vaccine. 2021 Oct 29;39(45):6700–11.

124. Reynolds CA, Finkelstein JA, Ray GT, Moore MR, Huang SS. Attributable healthcare utilization and cost of pneumonia due to drug-resistant streptococcus pneumonia: a cost analysis. Antimicrob Resist Infect Control. 2014 May 21;3(1).

125. Van Effelterre T, Moore MR, Fierens F, Whitney CG, White L, Pelton SI, et al. A dynamic model of pneumococcal infection in the United States: Implications for prevention through vaccination. Vaccine. 2010 May 7;28(21):3650–60.

126. Mitchell PK, Lipsitch M, Hanage WP. Carriage burden, multiple colonization and antibiotic pressure promote emergence of resistant vaccine escape pneumococci. Philosophical Transactions of the Royal Society B: Biological Sciences [Internet]. 2015 [cited 2021 Oct 27];370(1670). Available from: http://dx.doi.org/10.1098/rstb.2014.0342orviahttp://rstb.royalsocietypublishing.org.

127. Chan CH, McCabe CJ, Fisman DN. Core groups, antimicrobial resistance and rebound in gonorrhoea in North America. Sex Transm Infect. 2012 Apr;88(3):200–4.

128. Duan Q, Carmody C, Donovan B, Guy RJ, Hui BB, Kaldor JM, et al. Modelling response strategies for controlling gonorrhoea outbreaks in men who have sex with men in Australia. PLoS Comput Biol. 2021 Nov 1;17(11).

129. Findlater L, Mohammed H, Gobin M, Fifer H, Ross J, Geffen Obregon O, et al. Developing a model to predict individualised treatment for gonorrhoea: a modelling study. BMJ Open. 2021 Jun 25;11(6).

130. Fingerhuth SM, Bonhoeffer S, Low N, Althaus CL. Antibiotic-Resistant Neisseria gonorrhoeae Spread Faster with More Treatment, Not More Sexual Partners. PLoS Pathog. 2016 May 1;12(5).

131. Trecker MA, Hogan DJ, Waldner CL, Dillon JAR, Osgood ND. Revised simulation model does not predict rebound in gonorrhoea prevalence where core groups are treated in the presence of antimicrobial resistance. Sex Transm Infect. 2015 Jun 1;91(4):300–2.

132. Turner KM, Christensen H, Adams EJ, McAdams D, Fifer H, McDonnell A, et al. Analysis of the potential for point-of-care test to enable individualised treatment of infections caused by antimicrobial-resistant and susceptible strains of Neisseria gonorrhoeae: a modelling study. BMJ Open. 2017 Jun 1;7(6).

133. Whittles LK, White PJ, Didelot X. Estimating the fitness cost and benefit of cefixime resistance in Neisseria gonorrhoeae to inform prescription policy: A modelling study. PLoS Med. 2017 Oct 1;14(10).

134. Whittles LK, White PJ, Didelot X. Assessment of the Potential of Vaccination to Combat Antibiotic Resistance in Gonorrhea: A Modeling Analysis to Determine Preferred Product Characteristics. Clin Infect Dis. 2020 Oct 15;71(8):1912–9.

135. Xiridou M, Soetens LC, Koedijk FDH, Van Der Sande MAB, Wallinga J. Public health measures to control the spread of antimicrobial resistance in Neisseria gonorrhoeae in men who have sex with men. Epidemiol Infect. 2015 Mar 15;143(8):1575–84.

136. Xiridou M, Lugnér A, De Vries HJC, Van Bergen JEAM, Götz HM, Van Benthem BHB, et al. Cost-Effectiveness of Dual Antimicrobial Therapy for Gonococcal Infections Among Men Who Have Sex With Men in the Netherlands. Sex Transm Dis. 2016;43(9):542–8.

137. Zienkiewicz AK, Verschueren Van Rees N, Homer M, Ong JJ, Christensen H, Hill D, et al. Agent-based modelling study of antimicrobial-resistant Neisseria gonorrhoeae transmission in men who have sex with men: towards individualised diagnosis and treatment. Sex Health. 2019;16(5):514–22.

138. Bartsch SM, Huang SS, Wong KF, Slayton RB, McKinnell JA, Sahm DF, et al. Impact of delays between clinical and laboratory standards institute and food and drug administration revisions of interpretive criteria for carbapenem-resistant Enterobacteriaceae. J Clin Microbiol. 2016 Nov 1;54(11):2757–62.

139. Dalben MDF, Teixeira Mendes E, Moura ML, Rahman DA, Peixoto D, Dos Santos SA, et al. A Model-Based Strategy to Control the Spread of Carbapenem-Resistant Enterobacteriaceae: Simulate and Implement. Infect Control Hosp Epidemiol. 2016 Nov 1;37(11):1315–22.

140. Ho K wai, Ng W tong, Ip M, You JHS. Active surveillance of carbapenem-resistant Enterobacteriaceae in intensive care units: Is it cost-effective in a nonendemic region? Am J Infect Control. 2016 Apr 1;44(4):394–9.

141. Kardaś-Słoma L, Lucet JC, Perozziello A, Pelat C, Birgand G, Ruppé E, et al. Universal or targeted approach to prevent the transmission of extended-spectrum beta-lactamase-producing Enterobacteriaceae in intensive care units: A cost-effectiveness analysis. BMJ Open. 2017 Nov 1;7(11).

142. Lee BY, Bartsch SM, Wong KF, McKinnell JA, Slayton RB, Miller LG, et al. The Potential Trajectory of Carbapenem-Resistant Enterobacteriaceae, an Emerging Threat to Health-Care Facilities, and the Impact of the Centers for Disease Control and Prevention Toolkit. Am J Epidemiol. 2016 Mar 1;183(5):471–9.

143. Opatowski L, Opatowski M, Vong S, Temime L. A One-Health Quantitative Model to Assess the Risk of Antibiotic Resistance Acquisition in Asian Populations: Impact of Exposure Through Food, Water, Livestock and Humans. Risk Analysis. 2021 Aug 1;41(8):1427–46.

144. Pelat C, Kardaś-Słoma L, Birgand G, Ruppé E, Schwarzinger M, Andremont A, et al. Hand Hygiene, Cohorting, or Antibiotic Restriction to Control Outbreaks of Multidrug-Resistant Enterobacteriaceae. Infect Control Hosp Epidemiol. 2016 Dec 7;37(3):272–80.

145. Piotrowska MJ, Sakowski K, Lonc A, Tahir H, Kretzschmar ME. Impact of inter-hospital transfers on the prevalence of resistant pathogens in a hospital–community system. Epidemics. 2020 Dec 1;33.

146. Tahir H, Lopez-Cortes LE, Kola A, Yahav D, Karch A, Xia H, et al. Relevance of intra-hospital patient movements for the spread of healthcareassociated infections within hospitals - A mathematical modeling study. PLoS Comput Biol. 2021 Feb 3;17(2).

147. Toth DJA, Samore MH, Nelson RE. Economic Evaluations of New Antibiotics: The High Potential Value of Reducing Healthcare Transmission Through Decolonization. Clin Infect Dis. 2021 Jan 15;72(Suppl 1):S34–41.

148. Lee BY, Yilmaz SL, Wong KF, Bartsch SM, Eubank S, Song Y, et al. Modeling the regional spread and control of vancomycin-resistant enterococci. Am J Infect Control. 2013 Aug;41(8):668–73.

149. Lowden J, Miller Neilan R, Yahdi M. Optimal control of vancomycin-resistant enterococci using preventive care and treatment of infections. Math Biosci. 2014;249(1):8–17.

150. Suthar N, Roy S, Call DR, Besser TE, Davis MA. An individual-based model of transmission of resistant bacteria in a veterinary teaching hospital. PLoS One. 2014 Jun 3;9(6).

151. Wei Y, Kypraios T, O’Neill PD, Huang SS, Rifas-Shiman SL, Cooper BS. Evaluating hospital infection control measures for antimicrobial-resistant pathogens using stochastic transmission models: Application to vancomycin-resistant enterococci in intensive care units. Stat Methods Med Res. 2018 Jan 1;27(1):269–85.

152. Yahdi M, Abdelmageed S, Lowden J, Tannenbaum L. Vancomycin-resistant enterococci colonization-infection model: Parameter impacts and outbreak risks. J Biol Dyn. 2012 Mar;6(2):645–62.

153. Booton RD, Meeyai A, Alhusein N, Buller H, Feil E, Lambert H, et al. One Health drivers of antibacterial resistance: Quantifying the relative impacts of human, animal and environmental use and transmission. One Health. 2021 Jun 1;12.

154. Knight GM, Costelloe C, Deeny SR, Moore LSP, Hopkins S, Johnson AP, et al. Quantifying where human acquisition of antibiotic resistance occurs: A mathematical modelling study. BMC Med. 2018 Aug 23;16(1).

155. Mponponsuo K, Leal J, Spackman E, Somayaji R, Gregson D, Rennert-May E. Mathematical model of the cost-effectiveness of the BioFire FilmArray Blood Culture Identification (BCID) Panel molecular rapid diagnostic test compared with conventional methods for identification of Escherichia coli bloodstream infections. J Antimicrob Chemother. 2022 Feb 1;77(2):507–16.

156. Talaminos A, López-Cerero L, Calvillo J, Pascual A, Roa LM, Rodríguez-Baño J. Modelling the epidemiology of Escherichia coli ST131 and the impact of interventions on the community and healthcare centres. Epidemiol Infect. 2016 Jul 1;144(9):1974–82.

157. Kaufhold S, Yaesoubi R, Pitzer VE. Predicting the Impact of Typhoid Conjugate Vaccines on Antimicrobial Resistance. Clin Infect Dis. 2019 Mar 7;68(Suppl 2):S96–104.

158. Lo NC, Gupta R, Stanaway JD, Garrett DO, Bogoch II, Luby SP, et al. Comparison of Strategies and Incidence Thresholds for VI Conjugate Vaccines Against Typhoid Fever: A Cost-effectiveness Modeling Study. Journal of Infectious Diseases. 2018 Nov 10;218:S232–42.

159. Manore C, Graham T, Carr A, Feryn A, Jakhar S, Mukundan H, et al. Modeling and Cost Benefit Analysis to Guide Deployment of POC Diagnostics for Non-typhoidal Salmonella Infections with Antimicrobial Resistance. Sci Rep. 2019 Aug 2;9(1):11245.

160. Saad NJ, Bowles CC, Grenfell BT, Basnyat B, Arjyal A, Dongol S, et al. The impact of migration and antimicrobial resistance on the transmission dynamics of typhoid fever in Kathmandu, Nepal: A mathematical modelling study. PLoS Negl Trop Dis. 2017 May 5;11(5).

161. Doan TN, Kong DCM, Marshall C, Kirkpatrick CMJ, McBryde ES. Modeling the impact of interventions against Acinetobacter baumannii transmission in intensive care units. Virulence. 2016 Feb 17;7(2):141–52.

162. Tan MW, Lye DC, Ng TM, Nikolaou M, Tam VH. Mathematical model to quantify the effects of risk factors on carbapenem-resistant Acinetobacter baumannii. Antimicrob Agents Chemother. 2014;58(9):5239–44.

163. Wang X, Chen Y, Zhao W, Wang Y, Song Q, Liu H, et al. A data-driven mathematical model of multi-drug resistant Acinetobacter baumannii transmission in an intensive care unit. Sci Rep. 2015;5.

164. Qiu Z, Feng Z. Transmission dynamics of an influenza model with vaccination and antiviral treatment. Bull Math Biol. 2010 Jan;72(1):1–33.

165. Wessel L, Hua Y, Wu J, Moghadas SM. Public health interventions for epidemics: implications for multiple infection waves. BMC Public Health. 2011 Dec;11(S1).

166. Hughes J, Huo X, Falk L, Hurford A, Lan K, Coburn B, et al. Benefits and unintended consequences of antimicrobial de-escalation: Implications for stewardship programs. PLoS One. 2017 Feb 1;12(2).

167. Pham TM, Büchler AC, Voor in ‘t holt AF, Severin JA, Bootsma MCJ, Gommers D, et al. Routes of transmission of VIM-positive Pseudomonas aeruginosa in the adult intensive care unit-analysis of 9 years of surveillance at a university hospital using a mathematical model. Antimicrob Resist Infect Control. 2022 Dec 1;11(1).

168. Zacher B, Haller S, Willrich N, Walter J, Sin MA, Cassini A, et al. Application of a new methodology and R package reveals a high burden of healthcare-associated infections (HAI) in Germany compared to the average in the European Union/European Economic Area, 2011 to 2012. Eurosurveillance. 2019 Nov 14;24(46).

169. Changruenngam S, Modchang C, Bicout DJ. Modelling of the transmission dynamics of carbapenem-resistant Klebsiella pneumoniae in hospitals and design of control strategies. Scientific Reports 2022 12:1. 2022 Mar 9;12(1):1–16.

170. Kunkel A, Lewnard JA, Pitzer VE, Cohen T. Antimicrobial resistance risks of cholera prophylaxis for United Nations peacekeepers. Antimicrob Agents Chemother. 2017 Aug 1;61(8).
